# Prediction of therapeutic response of unresectable hepatocellular carcinoma to hepatic arterial infusion chemotherapy based on pretherapeutic MRI radiomics and Albumin-Bilirubin score

**DOI:** 10.1007/s00432-022-04467-3

**Published:** 2022-11-12

**Authors:** Yang Zhao, Fang Huang, Siye Liu, Lian Jian, Xibin Xia, Huashan Lin, Jun Liu

**Affiliations:** 1 Department of Radiology, Hunan Cancer Hospital, The Affiliated Cancer Hospital of Xiangya School of Medicine, Central South University, Changsha, 410006 Hunan People’s Republic of China; 2 Department of Interventional Therapy, Hunan Cancer Hospital, The Affiliated Cancer Hospital of Xiangya School of Medicine, Central South University, Changsha, 410006 Hunan People’s Republic of China; 3 Department of Infectious DiseaseThe Third Xiangya Hospital, Central South University, Changsha, 410013 Hunan People’s Republic of China; 4 Department of Pharmaceutical Diagnosis, GE Healthcare, Changsha, 410005 Hunan People’s Republic of China

**Keywords:** Hepatocellular carcinoma, Radiomics, Hepatic arterial infusion chemotherapy, Albumin-Bilirubin score, Nomogram, Therapeutic response

## Abstract

**Purpose:**

To construct and validate a combined nomogram model based on magnetic resonance imaging (MRI) radiomics and Albumin-Bilirubin (ALBI) score to predict therapeutic response in unresectable hepatocellular carcinoma (HCC) patients treated with hepatic arterial infusion chemotherapy (HAIC).

**Methods:**

The retrospective study was conducted on 112 unresectable HCC patients who underwent pretherapeutic MRI examinations. Patients were randomly divided into training (*n* = 79) and validation cohorts (*n* = 33). A total of 396 radiomics features were extracted from the volume of interest of the primary lesion by the Artificial Kit software. The least absolute shrinkage and selection operator (LASSO) regression was applied to identify optimal radiomic features. After feature selection, three models, including the clinical, radiomics, and combined models, were developed to predict the non-response of unresectable HCC to HAIC treatment. The performance of these models was evaluated by the receiver operating characteristic curve. According to the most efficient model, a nomogram was established, and the performance of which was also assessed by calibration curve and decision curve analysis. Kaplan–Meier curve and log-rank test were performed to evaluate the Progression-free survival (PFS).

**Results:**

Using the LASSO regression, we ultimately selected three radiomics features from T2-weighted images to construct the radiomics score (Radscore). Only the ALBI score was an independent factor associated with non-response in the clinical model (*P* = 0.033). The combined model, which included the ALBI score and Radscore, achieved better performance in the prediction of non-response, with an AUC of 0.79 (95% CI 0.68–0.90) and 0.75 (95% CI 0.58–0.92) in the training and validation cohorts, respectively. The nomogram based on the combined model also had good discrimination and calibration (*P* = 0.519 for the training cohort and *P* = 0.389 for the validation cohort). The Kaplan–Meier analysis also demonstrate that the high-score patients had significantly shorter PFS than the low-score patients (*P* = 0.031) in the combined model, with median PFS 6.0 vs 9.0 months.

**Conclusion:**

The nomogram based on the combined model consisting of MRI radiomics and ALBI score could be used as a biomarker to predict the therapeutic response of unresectable HCC after HAIC.

## Introduction

Hepatocellular carcinoma (HCC), the fourth leading cause of cancer death, is a common malignant tumor with more than 840,000 new cases worldwide each year (Bray et al. 2018). A comparative study of over 8,000 HCC cases showed that fewer than 10% of patients met the criteria of hepatectomy (Roayaie et al. 2015). For unresectable HCC, transcatheter arterial chemoembolization (TACE) is a well-established treatment. It is generally believed to be an effective treatment for preventing tumor growth and improving prognosis in unresectable HCC patients (2018; Shim et al. 2012). However, in the last few years, hepatic arterial infusion chemotherapy (HAIC) has been demonstrated to have fewer side effects and thus was considered to be superior to TACE in the treatment of unresectable HCC (He et al. 2017; Kudo et al. 2014). In addition, some studies have shown that HAIC might improve the median overall survival (OS) and disease-free survival (DFS) compared to sorafenib. Therefore, HAIC has been widely recommended as the first-line therapy for unresectable HCC patients in western countries (Choi et al. 2018; Lyu et al. 2018). Additionally, in several retrospective studies, the HAIC responders had significantly higher survival rates than HAIC non-responders (Miyaki et al. 2012; Nagano et al. 2011). Therefore, it is necessary to evaluate response to HAIC treatment as early as possible. According to Response Evaluation Criteria in Solid Tumors (RECIST), the conventional methods of assessing treatment response are imaging studies, including contrast-enhanced CT and MRI (Eisenhauer et al. 2009). These imaging studies are performed 1 month after each serial HAIC treatment. This means that the evaluation of therapeutic response can not be confirmed until at least the first course is completed. Therefore, to improve the prognosis and avoid hepatic function impairment in patients with HCC, predicting therapeutic response before deciding on HAIC protocol is of vital importance.

Radiomics has emerged as a new field in tumor treatment research in recent years (Gillies et al. 2016). It can transform massive image features into high-dimensional data that can be combined with clinical data, genetic information, and so on, to develop and validate various models using machine learning algorithms or artificial intelligence to help in medical decision-making (Aerts et al. 2014; Lambin et al. 2012; Mao et al. 2019a, b). In studies of HCC, radiomics has been used to forecast patients' prognoses and help make treatment choices (Li et al. 2016; Liu et al. 2021b; Simpson et al. 2015). In addition, the Albumin-Bilirubin (ALBI) score has been validated as an effective and objective marker of liver reserve function and a prognostic indicator after interventional therapy in middle and advanced-stage HCC patients (Chen et al. 2022; Ni et al. 2020). Thus, we hypothesized that an MRI-based radiomics score and ALBI score might be used to predict therapeutic response prior to HAIC treatment.

To our knowledge, the value of an MRI-based radiomics combined with ALBI scores in predicting the HCC’s therapeutic response after HAIC has not yet been investigated. Thus, the purpose of the current study was to construct and validate a novel nomogram based on a pretherapeutic MRI-based radiomics score combined with an ALBI score to predict the therapeutic response of unresectable HCC after HAIC.

## Materials and methods

### Patients

This single-center retrospective study was conducted in accordance with the Declaration of Helsinki and was approved by the research ethics committee of the Affiliated Cancer Hospital of Xiangya School of Medicine, Central South University, and the requirement for written informed consent was waived. According to the American Association for the Study of Liver Disease (AASLD) practice guidelines (Heimbach et al. 2018), 112 HCC patients were included in our study. The study population was screened from 281 HCC patients who received HAIC treatment in our hospital between November 2020 and October 2021, and their characteristics are shown in Table 1. The inclusion criteria were as follows: (1) diagnosed with unresectable HCC by our hospital's multidisciplinary team; (2) HCC cases with Barcelona Clinic Liver Cancer (BCLC) stage B or C; (3) received HAIC at our hospital; (4) performance status (PS) score < 2; and (5) Child–Pugh stage: A or B (7–8 points). Exclusion criteria were as follows: (1) HCC diagnosed with CT rather than MRI; (2) lost to follow-up or irregular follow-ups, not enough clinical data for assessing therapeutic response; (3) received HCC-related therapies before HAIC; (4) history of other cancers; (5) extrahepatic metastasis; and (6) diffuse-type HCC.

**Table 1:** Patient characteristics in the training and validation cohorts

Characteristics	Training cohort (*n* = 79)			Validation cohort (n = 33)		
	PR (*n* = 27)	PD + SD (*n* = 52)	*p*	PR (*n* = 11)	PD + SD (*n* = 22)	*p*
Gender			0.355			0.632
Male	23 (85.2%)	49 (94.2%)		8 (72.7%)	19 (86.4%)	
Female	4 (14.8%)	3 (5.8%)		3 (27.3%)	3 (13.6%)	
Age (y)	51.7 ± 13	50.8 ± 12.9	0.761	54.7 ± 8.2	53.9 ± 10.4	0.810
Maximum tumor size (mm)			1.000			0.701
> 100	18 (66.7%)	34 (65.4%)		8 (72.7%)	13 (59.1%)	
≤ 100	9 (33.3%)	18 (34.6%)		3 (27.3%)	9 (40.9%)	
BCLC stage			0.456			1.000
B	1 (3.7%)	6 (11.5%)		1 (9.1%)	3 (13.6%)	
C	26 (96.3%)	46 (88.5%)		10 (90.9%)	19 (86.4%)	
Tumor number (N)			0.318			1.000
1	11 (40.7%)	14 (26.9%)		4 (36.4%)	9 (40.9%)	
> 1	16 (59.3%)	38 (73.1%)		7 (63.6%)	13 (59.1%)	
PVTT type			0.456			1.000
None	1 (3.7%)	6 (11.5%)		2 (18.2%)	3 (13.6%)	
I	6 (22.2%)	14 (26.9%)		3 (27.3%)	1 (4.5%)	
II	11 (40.7%)	16 (30.8%)		5 (45.5%)	11 (50.0%)	
III	8 (29.6%)	15 (28.8%)		1 (9.1%)	6 (27.3%)	
IV	1 (3.7%)	1 (1.9%)		0 (0.0%)	1 (4.5%)	
Cause of disease			0.348			1.000
HBV	27 (100%)	48 (92.3%)		10 (90.9%)	20 (90.9%)	
Others^a^	0 (0%)	4 (7.7%)		1 (9.1%)	2 (9.1%)	
Child–Pugh class			1.000			0.150
A	23 (85.2%)	45 (86.5%)		11 (100.0%)	16 (72.7%)	
B	4 (14.8%)	7 (13.5%)		0 (0.0%)	6 (27.3%)	
DCP (mAU/ml)			1.000			0.678
≤ 1000	4 (14.8%)	7 (13.5%)		4 (36.4%)	5 (22.7%)	
> 1000	23 (85.2%)	45 (86.5%)		7 (63.6%)	17 (77.3%)	
ALT (U/L)			0.151			1.000
≤ 40	8 (29.6%)	7 (13.5%)		2 (18.2%)	5 (22.7%)	
> 40	19 (70.4%)	45 (86.5%)		9 (81.8%)	17 (77.3%)	
AST (U/L)			1.000			1.000
≤ 40	2 (7.4%)	4 (7.7%)		1 (9.1%)	2 (9.1%)	
> 40	25 (92.6%)	48 (92.3%)		10 (90.9%)	20 (90.9%)	
ALB (g/L)			0.245			0.314
≤ 35	3 (11.1%)	13 (25.0%)		1 (9.1%)	7 (31.8%)	
> 35	24 (88.9%)	39 (75.0%)		10 (90.9%)	15 (68.2%)	
TBIL (μmol/L)			0.915			0.900
≤ 20	12 (44.4%)	21 (40.4%)		7 (63.6%)	12 (54.5%)	
> 20	15 (55.6%)	31 (59.6%)		4 (36.4%)	10 (45.5%)	
PT (s)			0.591			0.105
≤ 13	10 (37.0%)	24 (46.2%)		9 (81.8%)	10 (45.5%)	
> 13	17 (63.0%)	28 (53.8%)		2 (18.2%)	12 (54.5%)	
AFP (ng/ml)			0.667			0.850
≤ 20	2 (7.4%)	7 (13.5%)		2 (18.2%)	2 (9.1%)	
20–200	3 (11.1%)	9 (17.3%)		2 (18.2%)	3 (13.6%)	
≥ 200	22 (81.5%)	36 (69.2%)		7 (63.6%)	17 (77.3%)	
ALBI	− 2.5 ± 0.5	− 2.3 ± 0.4	0.025	− 2.5 ± 0.4	− 2.3 ± 0.4	0.082

*DCP* des-γ-carboxy prothrombin, *ALT* alanine aminotransferase, *AST* aspartate aminotransferase, *ALB* albumin, *TBIL* total bilirubin, *PT* prothrombin time, *AFP* Alpha fetoprotein level, *PVTT* portal vein tumor thrombus, *ALBI* Albumin-Bilirubin score

^a^Including 4 case of HCV and 3 cases with unknown causes

**Fig. 1:**
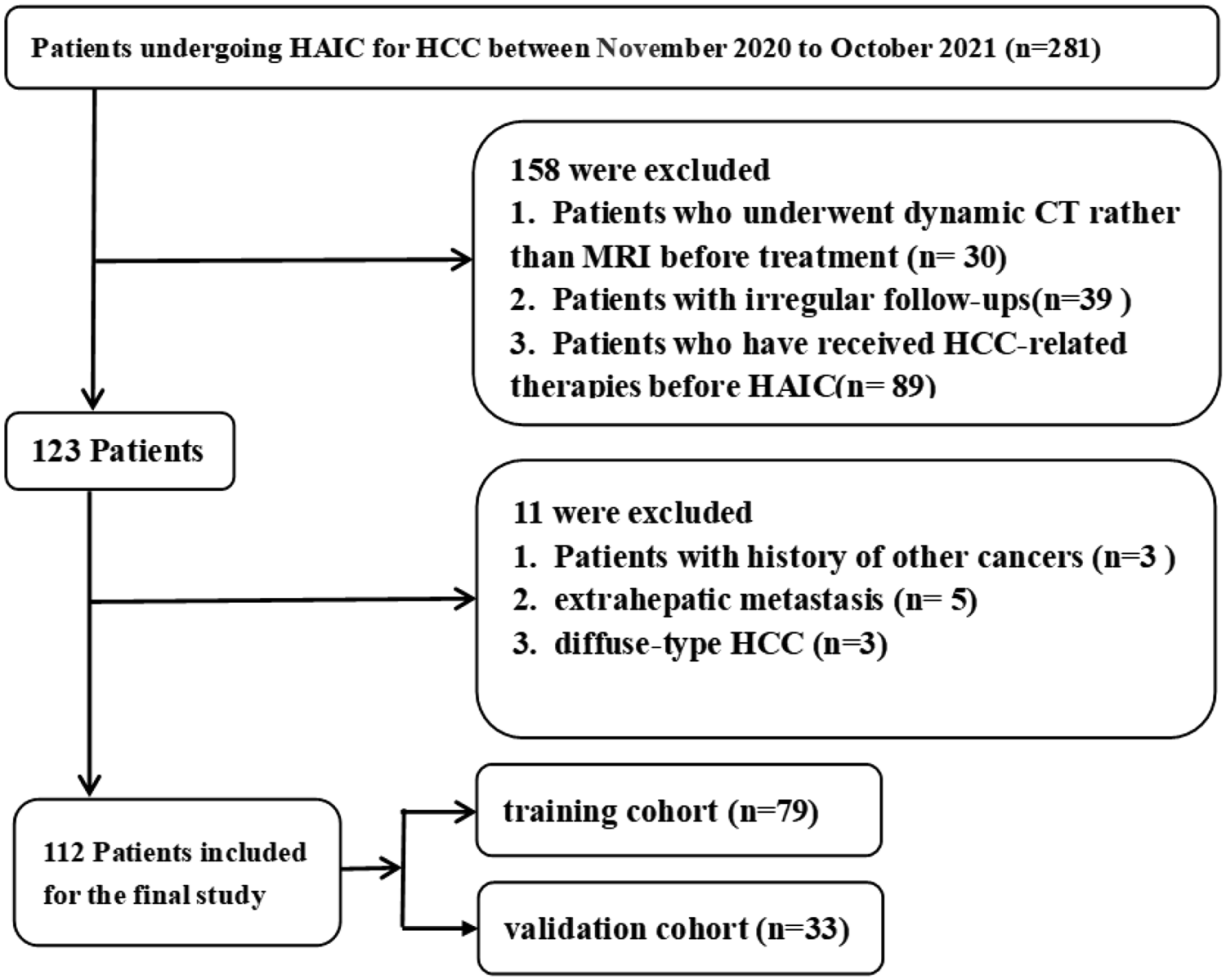
Flow chart for screening HCC patients treated with HAIC in our hospital

Finally, the patients were randomly divided into training and validation cohorts at a ratio of 7:3. A flow chart of patient selection is presented in Fig. 1.

### HAIC therapy procedure

All routine laboratory blood data were obtained within 3 days before HAIC. A femoral arterial puncture was performed in every cycle of treatment. A microcatheter was inserted into the feeding arteries of the HCC. The FOLFOX4 regimen was used via the hepatic artery: leucovorin 400 mg/m^2^, oxaliplatin 85 mg/m^2^, and fluorouracil 400 mg/m^2^ on the first day, and fluorouracil 2400 mg/m^2^ for 46 h. Repeat HAIC treatments were performed after every 3 weeks. Then, the therapeutic effect was assessed by imaging studies after every therapy cycle.

HAIC treatment could be combined with targeted therapy and immunotherapy.

Targeted therapies included Lenvatinib or Bevacizumab, and immunotherapies included Nivolumab or Atezolizumab.

### Assessment of response to HAIC and follow-up

The response to the course of HAIC treatment was evaluated by the RECIST criteria. The best imaging response (Miyaki et al. 2015) was assessed by experienced radiologists, who were uninformed about the treatment, using either a contrast-enhanced CT or MRI. All best imaging responses were assessed at least 3 weeks after the first HAIC treatment. Objective response rate (ORR) is defined as the rate of complete response (CR) plus partial response (PR). Disease control rate (DCR) is defined as the CR rate plus PR rate plus stable disease (SD) rate. PFS is defined from the day of initial HAIC treatment to the date of disease progression, death or the end day of the follow-up. In this study, we compared the HAIC treatment response between responders (CR + PR) and non-responders (PD + SD).

The follow-up ended in August 2022. Liver MR or CT imaging was performed every 3–4 weeks. Laboratory data, including the serum alpha-fetoprotein (AFP) level and liver function test results, were collected during each HAIC treatment cycle. The next treatment was determined by our hospital's multidisciplinary team (MDT). The ALBI score was calculated by the formula: ALBI score =  (lg bilirubin [μmol/L] × 0.66) − (albumin [g/L] × 0.085).

### MR imaging acquisitions

The preoperative MRI examination was performed with 3.0 T MRI scanner (Achieva; Philips Medical Systems, Best, the Netherlands) with 16 channels phased array coil serving as the receiver coil. The MRI protocol included: (1) a respiration-triggered turbo spin-echo (TSE) T2-w fat-suppressed axial imaging; TR = 3000 ms, TE = 70 ms, slice thickness = 5 mm, intersection gap = 1.1 mm, matrix = 320 × 280, and field of view (FOV) = 36 × 36 cm; (2) plain and contrast-enhanced T1-w imaging; T1-w 3D sequence (T1 high-resolution isotropic volume examination, THRIVE) was performed following the intravenous injection of Magnevist (Bayer Healthcare, Germany, 0.1 mmol/kg); TR = 4.1 ms, TE = 1.4 ms, slice thickness = 1 mm, matrix = 252 × 198, and FOV = 36 × 36 cm; 15 s acquisition time for one phase. In this study, only T2-w images were selected for radiomics analysis.

### Texture features extraction

Axial T2-w images (T2WI) were transferred into an open-source ITK-SNAP software (version 3.6.0, www. itksnap.org) for manual segmentation; the region of interest (ROI) on the MR images cannot be obtained automatically. As a result, an experienced radiologist (with more than 10 years of experience in abdominal radiology), blinded to the patients’ clinical status, reviewed the aforementioned MR images and manually traced the border of the lesions slice by slice to acquire a 2D ROI. Subsequently, the three-dimensional volume of interest (3D VOI) was constructed automatically based on the 2D ROIs of the intrahepatic lesion. Subsequently, the radiomics features from each VOI were extracted using in-house software (Artificial Intelligence Kit, A.K, GE Healthcare). A total of 396 radiomics features were extracted from MRI images, including (1) histogram features, such as entropy, energy, kurtosis, uniformity, and skewness; (2) form factor features, including compactness, surface area, sphericity, maximum 3D diameter, surface volume ratio, volume CC, spherical disproportion, and volume MM; (3) texture features, such as gray-level size zone matrix (GLZSM), gray-level co-occurrence matrix (GLCM), gray-level run-length matrix (GLRLM), and Haralick parameters.

### Feature selection and construction of RadScore

For the training cohort, mRMR (Max-Relevance and Min-Redundancy) was performed to eliminate the redundant and irrelevant features, and finally, 30 features were retained. Then LASSO (least absolute shrinkage and selection operator) regression was used to choose the optimized subset of features to construct the radiomics model (Gui and Li 2005; Huang et al. 2016). The Radscore was calculated by weighting the respective coefficients of linear combinations of the optimal radiological features selected from the training cohort to predict the non-response (PD + SD) with HAIC treatment for each patient. The area under the receiver–operator characteristic (ROC) curve (AUC) was used to assess the performance of the Radscore in both the training and validation cohort.

### Prognostic model construction

We used univariate analysis and multivariate regression to select optimal clinical factors. Then these significant factors were used to construct the clinical model by logistic regression. Lastly, these clinical independent risk factors and Radscore were utilized to build a combined model in the training cohort. The discriminative power of the model was evaluated by the area under the ROC curve with 95% confidence intervals. In addition, the accuracy, sensitivity, specificity, positive-predictive value (PPV), and negative-predictive value (NPV) for the clinical model, radiomics score, and combined model, were calculated in both cohorts. Then, a nomogram was established based on the most efficient model. Furthermore, the prediction accuracy of this nomogram model was explored using the calibration curve. And the decision curve analysis (DCA) was applied to assess the actual usefulness of the nomogram.

**Fig. 2:**
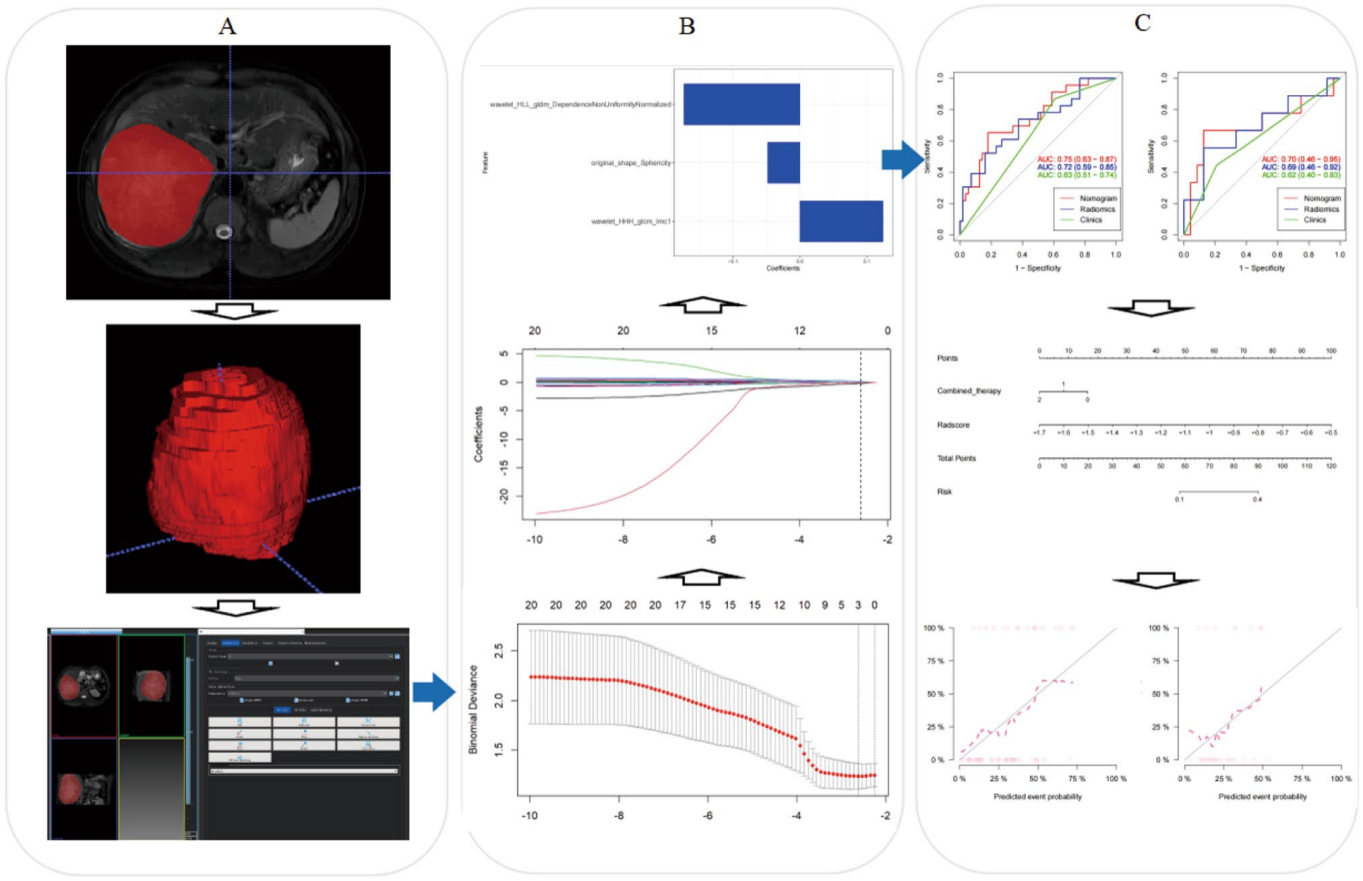
Workflow of the study: **A** Tumor segmentation on MR images. Radiomic feature extraction from VOI. **B** The LASSO regression was applied to identify optimal radiomic features and construct the Radscore. **C** The nomogram model was established based on the most efficient model. ROC and calibration curves were constructed to assess the model performance

Figure 2 shows the radiomics workflow of this study.

### Statistical analysis

The R3.5.1 software was used for statistical analyses. *P* < 0.05 was regarded as statistically significant. The Mann–Whitney *U*-test or independent-sample *t* test were used to compare the continuous variables. The most discriminating radiomics features were selected by LASSO regression. A radiomics signature was constructed via tenfold cross-validation based on the minimum criteria. The significant clinical factors were used to construct the clinical model via multivariate logistic regression analysis. The “rms” package program was applied to construct a nomogram and execute calibration curves. The calibration curve, ROC curve, and DCA were employed to assess the efficiency of the nomogram. Potential correlation of the combined model with PFS was evaluated using Kaplan–Meier analysis and log-rank test.

## Results

A total of 112 cases with confirmed unresectable HCC [median age, 53 years; interquartile range (IQR), 43–60 years; 13 women and 99 men] were included in our study, including 79 cases [median age, 52 years; interquartile range (IQR), 41–60 years; 7 women and 72 men] in the training and 33 cases [median age, 55 years; interquartile range (IQR), 47–60 years; 6 women and 27 men] in the validation cohort. The number of patients who presented with CR, PR, SD, and PD was 0 (0%), 38 (33.9%), 35 (31.2%), and 39 (34.8%), respectively. The ORR was 33.9%, and DCR was 65.2%. A total of 21 (18.7%) patients died before the end day of the follow-up. The differences in clinical characteristics between the PR and PD + SD groups in both cohorts are shown in Table 1. Of note, the number for targeted therapy and immunotherapy combined with HAIC was shown in Table 2. The proportion of combination therapies after HAIC in two cohorts showed no significant difference (*P* = 0.158). Hence, the influencing factor of treatment on therapeutic response was excluded, which make the statistical analysis in this study more reliable.

**Table 2:** Combined treatment with HAIC

	Training cohort (*n* = 79)	Validation cohort (*n* = 33)	*p* value
HAIC alone	11	9	0.158
HAIC + targeted therapy	21	10	
HAIC + targeted therapy + immunotherapy	47	14	

### Feature selection and rad-score establishment

**Fig. 3:**
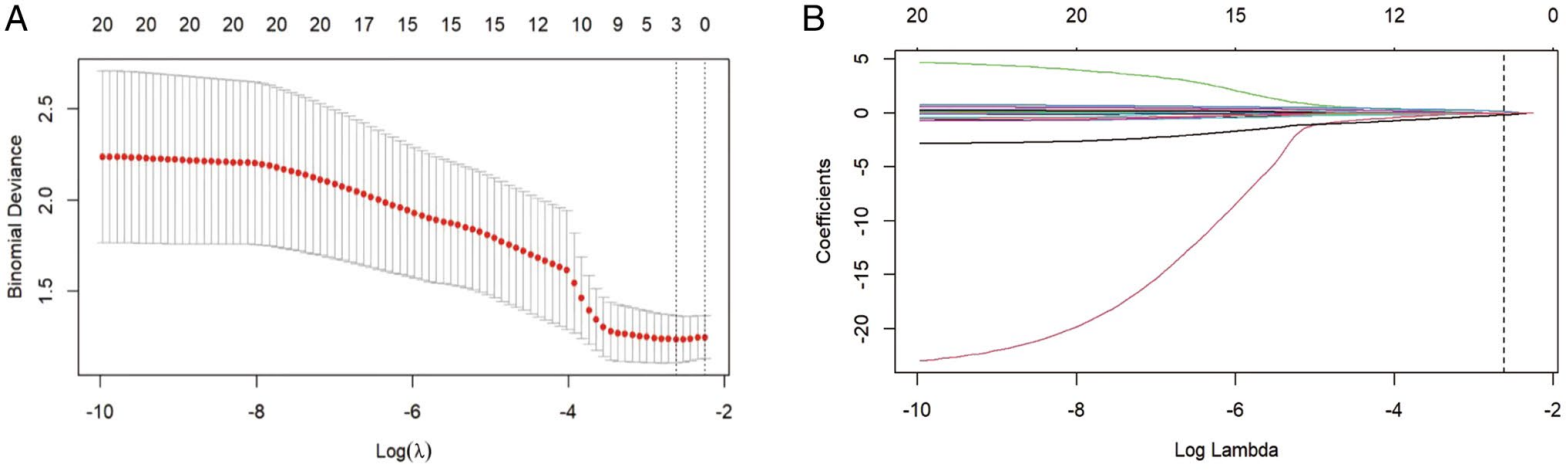
LASSO algorithm was used to choose optimal radiomic features to construct Radscore model: **A** mean square error path using tenfold cross-validation; **B** LASSO coefficient profiles of the radiomics features

For the 396 MRI radiomics features, the LASSO regression selected three significant radiomics features, namely, *wavelet_LLH_glcm_Correlation*,*wavelet_HHL_glcm_Correlation*, and *log_sigma_2_0_mm_3D_glszm_ZoneEntropy*were to construct radiomics model from the training cohort (Fig. 3). These features were used to calculate the Radscore using the following formula: Radscore = 0.637 × (Intercept) + 0.045 × wavelet_LLH_glcm_Correlation − 0.072 × wavelet_HHL_glcm_Correlation − 0.144 × log_sigma_2_0_mm_3D_glszm_ZoneEntropy.

The radiomics model based on the Radscore yielded an AUC of 0.70 (95% CI 0.58–0.82) in the training cohort and 0.69 (95% CI 0.51–0.88) in the validation cohort in predicting the SD + PD responses.

### Independent indicators of therapeutic response

Univariate analysis and multivariate logistic regression analysis showed that among all clinical features, only the ALBI score was an independent factor for therapeutic response (*P* = 0.033). The hazard ratio (HR) for the ALBI score was 3.69 (95% CI 1.10–12.30). Then, the clinical model was constructed based on the ALBI score to predict SD + PD responses, with an AUC of 0.68 (95% CI 0.55–0.81) in the training cohort and 0.71 (95% CI 0.51–0.91) in the validation cohort.

After combining the Radscore with the above clinic feature, the ALBI score and the Radscore were independent indicators of therapeutic response (*P* = 0.024 and 0.004). The HR for the ALBI score and the Radscore were 2.14 (95% CI 1.10–4.15) and 124.45 (95% CI 5.86–1347), respectively (Table 3).

**Table 3:** Logistic analysis of Radscore and clinical features for evaluation of HAIC response in training cohort

Characteristics	Univariate analysis		Multivariate analysis	
	*P*	HR (95% CI)	*P*	HR (95% CI)
Gender (male/female)	0.194	2.840 (0.586–13.746)		
Age (y) (< 60/ ≥ 60)	0.758	0.994 (0.958–1.031)		
Maximum tumor size (> 100 mm/ ≤ 100 mm)	0.909	0.944 (0.353–2.524)		
BCLC stage (B/C)	0.270	0.294 (0.033–2.584)		
Tumor number (N) (1/ > 1)	0.213	1.866 (0.698–4.983)		
PVTT (none/I/II/III/IV)	0.270	0.294 (0.033–2.584)		
HBV/others	0.989	0.775 (0.480–1.252)		
Child–Pugh class (A/ B)	0.869	0.894 (0.237–3.372)		
DCP (≤ 1000/> 1000)	0.264	0.647 (0.301–1.388)		
ALT (≤ 40/> 40 U/L)	0.088	2.706 (0.859–8.526)		
AST (≤ 40/> 40 U/L)	0.963	0.960 (0.164–5.607)		
ALB (≤ 35 /> 35 g/L)	0.155	2.666 (0.688–10.332))		
TBIL (≤ 20 /> 20 μmol/L)	0.728	1.180 (0.461–3.022)		
PT (≤ 13/> 13 s)	0.438	0.686 (0.264–1.779)		
AFP (≤ 20/20–200/ ≥ 200)	0.264	0.647 (0.301–1.388)		
ALBI score	0.033	3.686 (1.104–12.300)	0.024	2.140 (1.10–4.15)
Radscore	0.005	130.95 (4.25–3644.14)	0.004	124.45 (5.86–1347)

### The nomogram establishment

**Fig. 4:**
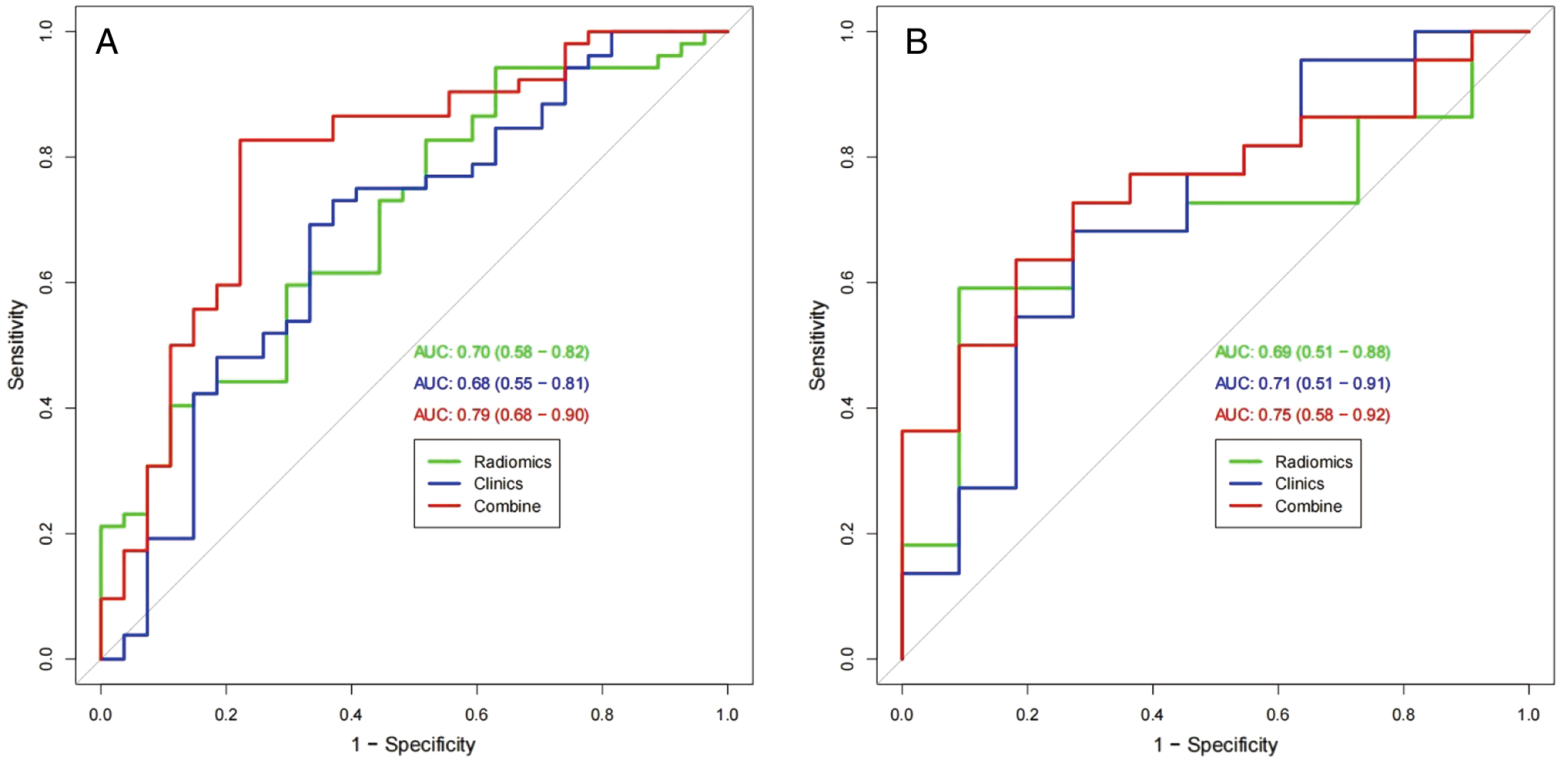
Receiver operating characteristic (ROC) curve for predicting the SD + PD response in the training cohort (**A**) and validation cohort (**B**)

**Fig. 5:**
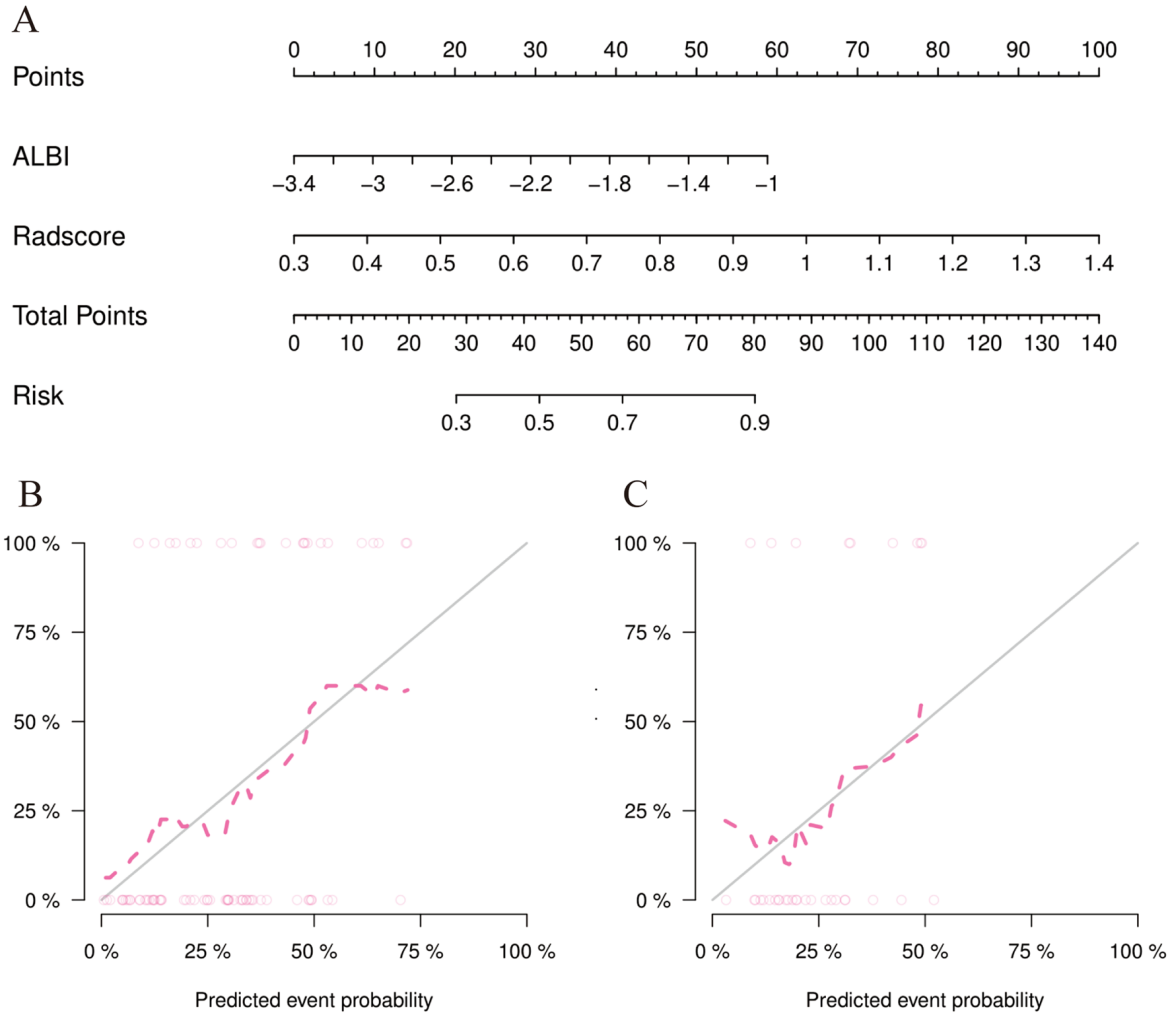
**A** The nomogram model based on Radscore and ALBI score for predicting SD + PD response of HCC to HAIC. **B** calibration curves of the nomogram model in the training cohort. **C** Calibration curves of the nomogram model in the validation cohort. The *X*-axis represents the predicted probability, and the *Y*-axis is the actual probability for the SD + PD response of HCC. Calibration curves indicate that there was a good agreement between the predicted probability and the actual state of treatment response

In the training cohort, the AUC of the combined model, the Radscore model, and the clinical model were 0.79, 0.70, and 0.68, respectively, while in the validation cohort, the AUC of these three models were 0.75, 0.69, and 0.71, respectively (Fig. 4). There were significant differences between the ROC of the combined model and the clinical model (*Z* = − 1.935, *P* = 0.046), but no significant differences were found between the ROC of the combined model and the Radscore model (*Z* = − 1.894, *P* = 0.058) using DeLong’s test. Furthermore, the combined model had the greatest accuracy (accuracy: 81.0%; specificity: 77.7%; sensitivity: 82.6%; NPV: 70.0%; PPV:87.7%) in predicting SD + PD responses (Table 4). Therefore, the nomogram was established based on the combined model (Fig. 5A).

**Table 4:** Accuracy and predictive value of three models

	Accuracy	95% CI	Sensitivity	Specificity	PPV	NPV
*Training cohort*						
Clinical model	0.696	0.582–0.794	0.730	0.629	0.791	0.548
Radiomic model	0.746	0.636–0.837	0.942	0.370	0.742	0.769
Combined model	0.810	0.706–0.889	0.826	0.777	0.877	0.700
*Validation cohort*						
Clinical model	0.636	0.451–0.795	0.590	0.727	0.812	0.470
Radiomic model	0.636	0.451–0.795	0.727	0.454	0.727	0.454
Combined model	0.696	0.512–0.844	0.727	0.636	0.800	0.538

CI, confifidence interval; NPV, negative-predictive value; PPV, positive-predictive value

The calibration curve of the nomogram’s probability in predicting SD + PD responses had a good agreement between the observed and predicted results in the training cohort and validation cohorts (Fig. 5B,C). The Hosmer–Lemeshow test showed no significant difference between ideal curves and calibration curves in the training (*P* = 0.519) and validation cohorts (*P* = 0.389), which represented a good fitting of the model.

**Fig. 6:**
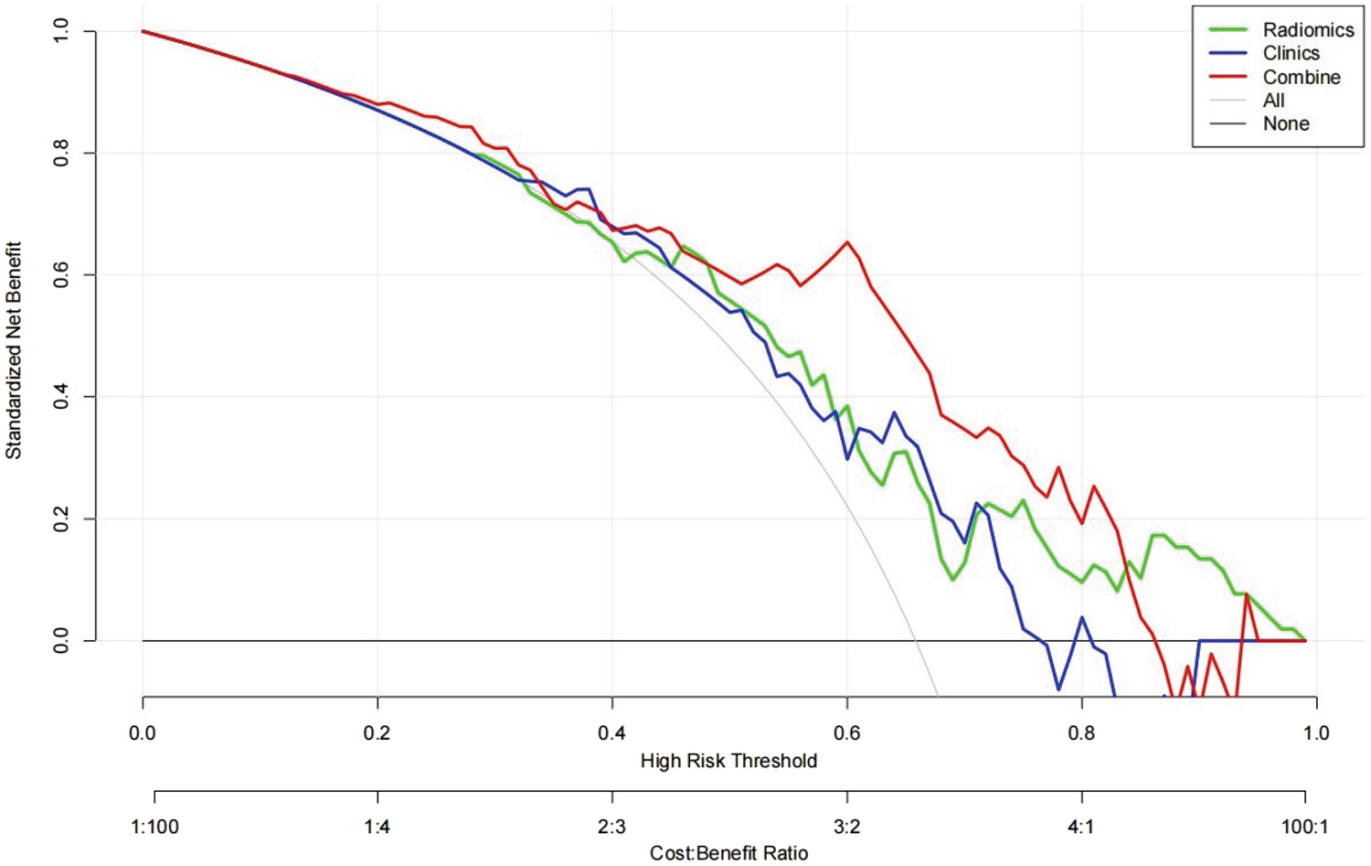
DCA curve for the nomogram model in the validation cohort. Compared to other models, the nomogram model demonstrated the highest area under the curve, and is the optimal decision that achieved the maximal net benefit in predicting SD + PD response of HCC after HAIC

The clinical utility of these three models was evaluated via DCA (Fig. 6). The nomogram showed a larger net benefit than did the radiomics and clinical models, which demonstrated the nomogram represented the best clinical utility for the prediction of therapeutic response of HCC after HAIC.

### Kaplan–Meier analysis for PFS

**Fig. 7:**
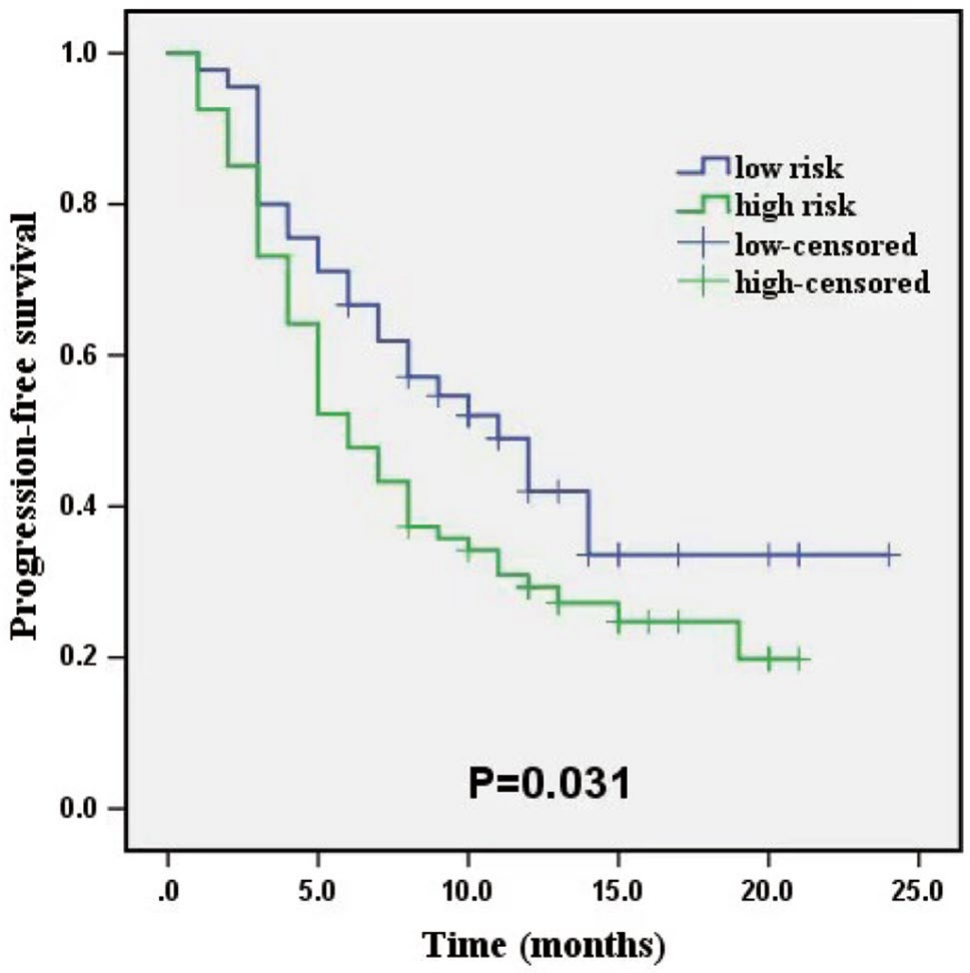
Kaplan–Meier curve of the low-risk and high-risk to nonresponse stratified by the combined model in the whole cohort

The Kaplan–Meier analysis was performed to assess the risk factor to PFS based on the aforementioned combined model in whole cohort. Using a cutoff value of 0.587 by ROC curve, patients were stratified into the low-score and high-score groups (low-score < 0.587 predicted HAIC-response, whereas high-score > 0.587 predicted HAIC-nonresponse). The high-score patients had significantly shorter PFS than the low-score patients (*P* = 0.031), with median PFS 6.0 vs 9.0 months (Fig. 7).

## Discussion

The current study evaluated the potential value of MRI-based radiomics combined with ALBI score in the prediction of the therapeutic response to HAIC in patients with unresectable HCC. We proved that intratumoral radiomics from pretherapeutic MRI imaging could improve the performance of clinical features in the prediction of therapeutic response. The combined model achieved an optimal predictive efficiency in patients with unresectable HCC. A nomogram combining radiomics features and ALBI score could efficiently separate those with SD + PD response from those with PR response in both training and validation cohorts. Meanwhile, the survival analysis also demonstrate that the patients with high-risk to non-response had significantly shorter PFS than the low-risk patients in the combined model. To the best of our knowledge, among all the related radiomics studies for HCC, our study was the first to establish a nomogram from the combined model to predict therapeutic response to HAIC in patients with HCC.

HAIC has been regarded as an effective treatment for advanced HCC in Asia. In several studies, HAIC responders have been proven to have longer survival rates than HAIC non-responders; thus, performing HAIC before administering molecular targeted therapy is recommended. Clinical features and tumor markers were used to predict the therapeutic response to HAIC in most of the previous studies; one of which reported that the early change of tumor markers and the imaging assessment 2 weeks after HAIC treatment were useful in predicting therapeutic response and prognosis (Miyaki et al. 2015). Yamamoto et al. (2020) suggested that the combination of optimal cutoff values for the AFP ratio and the DCP ratio after the initiation of HAIC enabled the prediction of non-responders and improved prognosis in patients with advanced HCC. Compared with these biomarkers, radiomics may serve as a relatively inexpensive and non-invasive biomarker as it has the potential to provide a more specific tumor characterization and guide clinicians in individualized treatment planning before therapy.

In our study, a nomogram combined with MRI radiomics and ALBI score showed remarkable differences between PR and SD + PD groups. Furthermore, radiomics score was identified by the univariate and multivariate logistic regression models as an independent predictor of non-response. In a previous study, VWF:Ag/ADAMTS13:AC ratio was used as a biomarker of treatment response in HCC patients before the initiation of HAIC treatment, and ROC curves were used to evaluate predictive efficiency, yielding an AUC of 0.715 (Takaya et al. 2020). However, our study showed that the radiomics-based nomogram in the training group had a higher AUC (AUC of 0.79). Therefore, we believe that, compared with clinical features, radiomics score may be a more powerful predictor. The nomogram based on a combined model in our study can serve as an adaptive strategy to improve the prognosis of HCC patients.

Currently, clinical MR scans are routinely acquired for diagnosis and staging in HCC. These MRI images contain important information about tumor heterogeneity, including biological characteristics determined by underlying micro-level components of the tumor tissue (Gillies et al. 2016; Rogers et al. 2020). Based on previous research, the calculation of radiomics features from T2WI imaging may be more accurate than from T1WI and enhanced T1WI, and can be further utilized as a reliable indicator for objective and high throughput image analysis (Liu et al. 2021a, b). Therefore, our study analyzed radiomics features from T2W images to predict the therapeutic response after HAIC.

In previous studies of HCC, radiomics obtained from pretreatment CT was thought to be a good predictor of the HCC treatment response after TACE, which could prevent unnecessary treatment (Park et al. 2017). Another study suggested that MRI-based radiomics may predict the prognosis of HCC treated with TACE combined with microwave ablation (Liu et al. 2021b). CT imaging radiomics was also useful for stratifying patients with HCC to determine appropriate treatment options between liver resection and TACE (Li et al. 2016). All relevant studies showed that radiomics has additional value to clinical features in predicting therapeutic response and prognosis of HCC. The results of our study indicated that the Radscore was an independent risk factor obviously related to the non-response of HCC to HAIC. Moreover, the HR of the Radscore was considerably higher than that of clinical features, which suggests that Radscore may be more helpful than the clinical characteristics in predicting the therapeutic response of unresectable HCC after HAIC.

In recent years, The ALBI score, a scoring system based solely on serum levels of albumin and total bilirubin, has been proposed as a simple and objective model to assess the liver reserve function in patients with HCC (Johnson et al. 2015). The elevation of serum bilirubin and reduction of albumin level usually indicates liver injury. Meanwhile, the reduction of albumin level is also associated with worse nutritional status of the body, leading to a decrease in immune function and, subsequently, tumor recurrence. HCC patients with higher ALBI scores have poorer liver reserve function, lower grade tumor biological behavior, worse systemic condition, and thus a worse prognosis. Our study has found that the higher ALBI score was an independent risk factor for non-response after the HAIC. The calibration curve of our nomogram based on the combined model (ALBI score and Radscore) verified that the predictive probability of the model fitted well with the actual therapeutic response, proving that the model has high accuracy. Therefore, the nomogram in this study can guide the prediction of the treatment response in HCC patients after HAIC.

This study has several limitations. First, selection bias might exist because of the retrospective design in our study. Second, although our cohort remains one of the largest in terms of radiomics features and interventional treatment of HCC, the patients were enrolled from a single institution with limited sample size, so the conclusions need to be verified by further prospective studies. Third, our study conducted image segmentation manually; an automated method for image segmentation may provide greater stability (de Hoop et al. 2009). Fourth, feature extraction was based on a single T2WI image for analysis in this study, but in practice, it is convenient for the clinician to extract radiomics features for analysis.

## Conclusion

In conclusion, the nomogram based on the combined model that incorporated MRI-based radiomics features and ALBI score exhibited favorable performance in predicting the response of unresectable HCC to HAIC. The pretherapeutic MRI-based radiomics score could be used as a non-invasive biomarker to help clinicians make reasonable clinical decisions, thereby avoiding the overtreatment of patients with HCC.

## Data Availability

The data used to support the fndings of this study are available from the corresponding author upon request.
